# Point-of-care ultrasound as a competency for general internists: a survey of internal medicine training programs in Canada

**Published:** 2016-10-18

**Authors:** Jonathan Ailon, Ophyr Mourad, Maral Nadjafi, Rodrigo Cavalcanti

**Affiliations:** 1 General Internal Medicine & Palliative Care, Saint Michael’s Hospital, Toronto, ON; 2 General Internal Medicine, Saint Michael’s Hospital, Toronto, ON; 3 General Internal Medicine, North York General Hospital, Toronto, ON; 4 General Internal Medicine, Toronto Western Hospital, Toronto, ON

## Abstract

**Background:**

Point-of-care ultrasound (POCUS) is increasingly used on General Internal Medicine (GIM) inpatient services, creating a need for defined competencies and formalized training. We evaluated the extent of training in POCUS and the clinical use of POCUS among Canadian GIM residency programs.

**Method:**

Internal Medicine trainees and GIM Faculty at the University of Toronto were surveyed on their clinical use of POCUS and the extent of their training. We separately surveyed Canadian IM Program Directors and Division Directors on the extent of POCUS training in their programs, barriers in the implementation of POCUS curricula, and recommendations for POCUS competencies in IM.

**Results:**

A majority of IM trainees (90/118, 76%) and GIM Faculty (15/29, 52%) used POCUS clinically. However, the vast majority of resident (111/117, 95%) and GIM Faculty (18/28, 64%) had received limited training. Of the Program Leaders surveyed, half (9/17, 53%) reported POCUS clinical use by their trainees; however only one quarter (4/16, 25%) reported offering formal curricula. Most respondents agreed that POCUS training should be incorporated into IM residency curricula, specifically for procedural guidance.

**Conclusions:**

A considerable discrepancy exists between the clinical use of POCUS and the extent of formal training among Canadian IM residents and GIM Faculty. We propose that formalized POCUS training should be incorporated into IM residency programs, GIM fellowships, and Faculty development sessions, and identify POCUS skills that could be incorporated into future IM curricula.

## Introduction

Point-of-care ultrasound (POCUS) refers to ultrasonography performed in real-time by the care provider at the patient’s bedside.^1^ POCUS has been demonstrated to improve diagnostic and procedural accuracy and improve patient care in cardiology,^2^–^4^ intensive care,^5^,^6^ rheumatology,^7^,^8^ respirology,^4^,^9^ endocrinology,^1^ and nephrology.^10^,^11^ Other clinical specialties such as Emergency Medicine, Critical Care, and Trauma Surgery have successfully established curricula to train residents to perform bedside diagnoses and procedures under ultrasound guidance.^1^,^12^,^13^ General Internists practicing in inpatient settings have also increasingly adopted the use of POCUS as an aid in clinical assessment and procedural guidance.^1^

Evidence suggests that clinicians can acquire “focused” ultrasound skills with directed training.^3^,^14^–^19^ POCUS has the potential to improve diagnostic accuracy by allowing the collection of more precise and timely clinical data, as well as increasing the procedural success rate and patient safety for procedures such as central vascular access, thoracentesis, paracentesis, and arthrocentesis.^6^,^20^–^26^

In the CanMEDS 2015 Patient Safety and Quality Improvement Expert Working Group Report, POCUS guidance is cited as one of the potential skills, or competencies, in residency training to improve safety in diagnostic and therapeutic procedures.^27^ However, there are a paucity of published guidelines or formal curricula for POCUS training in Internal Medicine (IM) programs.^28^,^29^ To the best of our knowledge, no studies have looked at the current state of clinical use of POCUS by Canadian residents and General Internal Medicine (GIM) Faculty. If the clinical use of POCUS has outpaced formal training on its safe applications, ultrasound studies performed by inexperienced users may result in harm to patients from inaccurate diagnoses, unnecessary additional tests, and procedural complications.^1^ In support of this concern, the Canadian Association of Radiologists developed a position statement in 2013 on POCUS asserting that, “Sonography equipment in the hands of an operator who is not well versed in the specific scope of examinations that are to be performed, has an increased likelihood of being more harmful than beneficial.”^30^ This study aimed firstly to identify the prevalence of POCUS use amongst IM residents and GIM faculty in Canada. Secondly, we identified the amount of formal training that respondents had received. Subsequently, we examined for discrepancies between the amount of formal training and the current clinical use of POCUS in Canada due to the implications of inadequate training on the unsafe use of POCUS in clinical care. Lastly, we identified potential barriers to the implementation of POCUS curricula in Canadian IM programs.

In this aim, we conducted two local and one national survey. The local surveys, conducted at the University of Toronto, aimed to establish the extent of clinical use and the level of POCUS training among IM residents and GIM faculty in Canada. Respondents were also asked their opinions on POCUS skills that would be valuable to the clinical practice of internists. With the national survey, we examined the current use of and training for POCUS in Canadian IM residency programs and aimed to understand potential barriers to the implementation of POCUS curricula in these programs. All three surveys examined for potential discrepancies between the formal training on POCUS and the current clinical use of POCUS.

## Methods

### Survey development

In the development of the three surveys (two local and one national), a panel was established at the University of Toronto consisting of one GIM Division Head, one GIM Fellowship Program Director, and one IM resident. All panel members had attended formal training in POCUS and were involved in ultrasound education. The main objectives of these surveys were to firstly to identify the current training on POCUS, the current clinical use of POCUS by IM residents and GIM staff. Secondly, this study aimed to acquire respondent suggestions on potential ultrasound skills, or competencies relevant to IM as well as their opinions on effective educational models for POCUS training. Questions were developed collectively by the panel and were reviewed for clarity and content by two GIM Faculty members who were independent of this study. No formal validation of these surveys was performed. The three surveys were approved through the Institutional Ethics Review Board. All survey responses were kept confidential and there were no monetary incentives to participate.

### Local surveys

We conducted an anonymous electronic survey of 194 IM residents in post-graduate years (PGY) 1 – 4 at the University of Toronto regarding their use of and training in POCUS (Appendix A). A similar electronic survey was sent to 58 GIM Faculty at the University of Toronto (Appendix B). The surveys were distributed using online survey software (fluidsurvey.com). The invitations to complete these online surveys were sent twice to all potential participants. These surveys included 16 questions and surveyed demographics, previous POCUS experiences, previous sonographic training, self-reported confidence in performing POCUS studies, interest in POCUS training, and perceived relevance of POCUS in IM. The surveys also solicited respondents’ opinions regarding the optimal time in residency to introduce POCUS training (which year of training), the preferred format of teaching (combined didactic and practical sessions, self-teaching and supervised tutorials, on-line teaching module, or other), and a list of POCUS skills that would be most relevant to clinical practice in inpatient IM. These two surveys differed in that the resident survey identified the residents’ level of training and the clinical services where the residents had used POCUS. The Faculty survey identified the number of years since FRCP certification and specifically asked Faculty how they would prefer to receive further training in POCUS (a self-directed online module, a local weekend of didactic and practical training sessions, a weekend course at a centre of excellence, or other).

### Canadian GIM programs survey

Leaders in GIM from across Canada were invited to participate in a survey over a 2-month period in 2011, including GIM Program Directors, GIM Division Directors, and Core Internal Medicine Program Directors. The survey assessed the extent and nature of POCUS training in their respective programs (Appendix C). The survey consisted of 8 questions and one open-text comment section and assessed respondent academic position, whether formal POCUS curricula were incorporated into their IM residency or GIM fellowship programs, the specific usage of POCUS by their trainees, the amount and format of POCUS training in their programs (didactic teaching, ultrasound images/videos, hands on training with simulators or patients, case logs, or other), their opinion on whether POCUS should be incorporated into IM residency and GIM fellowship programs, whether they have dedicated POCUS equipment, and potential barriers to the implementation of POCUS curricula (lack of machines, lack of trained Faculty, lack of interest/support, formal radiology studies readily available, financial reasons, opposition from other US-trained physicians, concerns that POCUS requires a long period of training, or other). The survey was distributed using online survey software (surveymonkey.com). The survey invitations were sent twice to all potential participants.

### Statistical analysis

All data were extracted from the online survey software into Microsoft Excel (2011). All data were summarized using descriptive statistics.

## Results

### Local surveys

Out of 194 IM residents surveyed, 118 (61%) responded. A majority of respondents were 25–29 years of age (89/117, 76%) with similar participation from both genders. Most respondents were in PGY1 and PGY2 (79/118, 67%). Twenty-nine GIM Faculty members responded (50%). The majority were senior Faculty who had obtained certification more than 10-years earlier (20/29, 69%) (Table 1).

While a minority of residents (16/118, 14%) had performed 10 or more ultrasound procedures independently, many (53/118, 45%) had witnessed ultrasounds performed and had brief exposure with an expert supervisor. Twenty-seven residents (23%) had only witnessed ultrasonography previously. Most residents used POCUS during critical care rotations. In contrast, only 2 GIM Faculty (7%) indicated that they had used ultrasound independently more than 10 times (Table 2).

Only 5% of residents (6/117) and 36% of GIM Faculty (10/28) reported formal training in general or specific ultrasound skills, while 32% of residents (37/117) and 14% of GIM Faculty (4/28) reported informal training in performing specific POCUS-guided procedures. Most residents (92/118, 78%) and GIM Faculty (18/28, 64%) reported a lack of comfort in using POCUS for procedures. Most residents (74/117, 63%) and half of the GIM Faculty (14/28, 50%) reported having received no training on POCUS (Table 2).

For individuals who used POCUS clinically, the most common reported applications included: central line insertion (residents 76%, GIM Faculty 42%) assessment of ascites for paracentesis (residents 67%, GIM Faculty 68%); and assessment of pleural effusion for thoracentesis (residents 59%, GIM Faculty 63%). Responses on these clinical uses of POCUS were similar between IM residents and GIM Faculty (Table 3).

The vast majority of residents (115/116, 99%) and GIM Faculty (28/29, 97%) felt that POCUS diagnostic and procedural skills were relevant to IM. The POCUS applications that were identified by respondents as being most relevant to IM were: central line insertion (residents 92%, GIM Faculty 86%); assessment of pleural effusion for thoracentesis (residents 89%, GIM Faculty 97%), and assessment of ascites for paracentesis (residents 85%, GIM Faculty 97%). Responses on these suggested applications were similar between IM residents and GIM Faculty for their top three selections (Table 4).

As assessed with a Likert scale, all residents (115/115, 100%) and most GIM Faculty (28/29, 97%) were either ‘somewhat interested’ or ‘very interested’ in including POCUS training in IM residency training. The majority of residents (101/117, 86%) and GIM Faculty (25/29, 89%) reported that a combined didactic and hands-on curriculum would be the most effective educational course model for POCUS training. A minority of respondents selected self-teaching and supervised tutorials or on-line teaching modules. Other respondents, in an open-text field, suggested a procedural rotation mixed with POCUS teaching from a radiologist or practical teaching with or without online modules (Table 5).

The majority of residents (95/116, 82%) and GIM Faculty (15/59, 52%) felt that POCUS training should be implemented starting in PGY1. Regarding the optimal way to incorporate POCUS training into the existing residency curriculum, most residents favoured POCUS teaching as part of academic half-days (59/118, 50%) while others preferred a dedicated weekend or one-week course on POCUS (49/118, 42%).

### Canadian GIM programs survey

Of the GIM Program Leaders surveyed, 17 of 32 (53%) responded consisting of: Core IM Program Directors (18%); GIM Division Heads (47%); GIM Fellowship Program Directors (24%); and two participants that held dual appointment (12%).

Among respondents to the national survey, 53% (9/17) reported that they had incorporated POCUS into their training programs. Of these respondents, the principle uses of POCUS was for POCUS guided vascular access (9/9, 100%), POCUS guided thoracentesis (6/9, 67%), and POCUS guided paracentesis (5/9, 56%) (Table 6).

All GIM Program Leaders agreed that POCUS training should be incorporated into GIM training Programs (17/17, 100%). However, several barriers to the successful implementation of POCUS were identified including: a lack of Faculty trained in POCUS (13/15, 87%); a lack of access to ultrasound equipment (7/15, 47%); and financial limitations (6/15, 40%) (Table 7).

## Discussion

In the 2013 position statement on the use of POCUS, the Canadian Association of Radiologists asserts their concern that ultrasound use by inexperienced providers may portend harm to patient care.^30^ In our study, most Internal Medicine trainees (76%) and GIM Faculty (52%) used POCUS clinically. However, the vast majority of residents (95%) and GIM Faculty (64%) had received none or only informal training on POCUS. This identifies a considerable gap between the education on POCUS and its clinical use. Without the implementation of thoughtful curricula on the safe application of POCUS within the scope of IM, ongoing clinical use of POCUS may portend harm to patients.

While the CanMEDS 2015 Patient Safety and Quality Improvement Expert Working Group Report lists ultrasound guidance as a potential competency to ensure patient safety and quality,^27^ there are no recommendations on the specific POCUS skills that should be targeted. Based on the two local surveys, there is a consensus in the respondents’ recommendations on POCUS competencies for internists including procedural guidance for central lines and in the assessment for ascites and pleural effusions and POCUS-guidance for a thoracentesis and paracentesis. These identified competencies are similar to previously reported Canadian consensus-based recommendations made by a panel of 13 ultrasound content experts.^31^ These suggested competencies have also been shown to improve procedural success rates and patient safety.^6^,^20^–^25^ The respondents suggested that POCUS curricula would be best delivered during an academic half-day starting in PGY1 using a combination of didactic and hands-on training sessions.

Of the Canadian GIM residency and fellowship Program Leaders that responded to this survey, all agreed that POCUS should be integrated into residency and fellowship programs. Even so, only 25% of these Program Leaders report offering formal training for POCUS. For sites where Program Leaders endorsed the clinical use of POCUS, the top three POCUS applications were procedural guidance for central lines and in the assessment for ascites and pleural effusions and POCUS-guidance for a thoracentesis and paracentesis. These clinical uses parallel the aforementioned recommended POCUS competencies in IM programs. Perceived barriers to overcome for the introduction of POCUS in IM training include training the Faculty, improving access to ultrasound equipment, and discovering innovative ways to fund POCUS training within each training program.

The findings from our study have several potential limitations. The two local surveys that were administered to the IM residents and GIM staff had modest response rates and are subject to sampling bias.^32^ Further, there is the possibility that residents and GIM Faculty with documented clinical use of POCUS may overestimate the extent of their formal training, subjecting this study to potential response bias. Based on the small sample size, and with the aim to preserve anonymity of respondents, we were unable to correlate the amount of reported training in POCUS of an individual respondent with the extent of their clinical use. As such, we were unable to determine if there was an association between the amount of POCUS training and the individual respondents’ comfort or clinical usage of POCUS. Furthermore, the local surveys sent to the residents and GIM Faculty differed on questions related to demographics. As such, there is the possibility of error in comparing data between these two surveys. To limit this potential source of error, comparisons between these surveys were only made for identical questions. Lastly, these surveys were administered locally at the University of Toronto and the findings may not generalize to other Canadian IM residents’ or GIM Faculty members’ experiences with POCUS.

The national survey included responses from the majority of IM Program Leaders in Canada [17/32]. Nonetheless, the number of respondents is small and it is difficult to determine whether these data accurately reflect the current usage and training for POCUS across Canada. In addition, we surveyed GIM Program Directors, GIM Division Directors, and Core Internal Medicine Program Directors. As such, it is possible that we received multiple responses from a single IM program. Due to the anonymity of data collection, we did not determine the respondents’ University affiliations and we were unable to account for this. The low response rate and the chance of multiple data from one program create the risk of sampling bias.^32^ In particular programs without POCUS curricula may not have participated in the survey, which would overestimate the prevalence of POCUS usage and teaching in IM programs in Canada. The findings of both the local and national surveys relate to the IM education system and the practice of General Internists in Canada, which is largely hospital-based. These findings may not generalize to other countries such as the United States where Internal Medicine has a larger role in ambulatory primary care. Lastly, this study provides a representation of the prevalence of POCUS in clinical use and the extent of POCUS training at the time of the study. POCUS is rapidly evolving within the medical community and a follow-up study would help elucidate changes in our findings.

Despite these limitations, this study highlights several important issues. There is an increasing need for formal training on POCUS within IM programs. IM competencies for ultrasound training should be well defined and focus on targeted clinical assessment skills and bedside procedures, relevant to the scope of practice of an Internist. More research is needed to establish a competency-based training framework and to develop validated assessment tools.

Following the findings of this study, the IM program at the University of Toronto has developed and launched a competency-based curriculum to teach focused diagnostic and procedural POCUS skills to IM trainees. This curriculum includes on-line modules followed by hands-on training with direct observation. Trainees will be able to electronically log POCUS studies and receive feedback on their sonographic skills and diagnostic accuracy. Residents subsequently undergo a structured standardized assessment to evaluate POCUS competency in specific competencies.

### Conclusions

The use of POCUS by inexperienced and untrained users may portend harm to patients while, if used properly, holds great potential to deliver clinical benefits to patients. We explored the current use of POCUS, and the status of POCUS training in IM. This study identifies a considerable gap between the current education on the safe applications of POCUS and its clinical use. We have demonstrated a desire amongst residents, GIM Faculty, and IM Program Leaders for formal training to be incorporated into Canadian IM residency and fellowship programs and GIM Faculty development sessions. Based on respondent input, we have outlined a list of POCUS procedural applications relevant to IM. The implementation of specific POCUS curricula within a defined scope of practice represents a key opportunity to improve clinical training in IM with the aim to improve patient care and safety.

## Figures and Tables

**Table 1:** Characteristics of IM residents (*n* = 117) and GIM faculty (*n* = 29)

Internal Medicine Resident Age	*n* (%)
19–24	3 (3)
25–29	89 (76)
30–35	24 (21)
35–39	0 (0)
>40	1 (1)

**Internal Medicine Resident Gender**	

Male	54 (46)
Female	63 (54)

**GIM Faculty Time Since FRCP Certification**	

1–5 years	3 (10)
5–10 years	6 (21)
10–20 years	13 (45)
> 20 years	7 (24)

**GIM Faculty Gender**	

Male	19 (66)
Female	10 (29)

**Table 2:** Internal medicine resident and GIM faculty responses on clinical use of POCUS, amount of POCUS training, and comfort in use of POCUS for procedures

	*n* (%)	
	Residents *n* =118	Faculty *n* =29
**Use of POCUS for Diagnostic Assessments and Procedures**		
Performed greater than 10 independent POCUS assessments	16 (14)	2 (7)
Witnessed many POCUS assessments and performed greater than 5 independent scans	21 (18)	8 (28)
Witnesses several assessments and performed POCUS with supervision	53 (45)	5 (17)
Witnesses but never performed POCUS assessments	27 (23)	8 (28)
Never witnessed or used an ultrasound machine	1 (1)	6 (21)

**Amount of Training in POCUS**	***n*** **=117**	***n*** **=28**
Received formal general POCUS training	2 (2)	2 (7)
Received formal training in specific POCUS assessments or procedures	4 (3)	8 (28)
Received informal training in specific POCUS assessments or procedures	37 (32)	4 (14)
No training in POCUS	74 (63)	14 (48)

**Comfort in Use of POCUS for Procedures**	***n*** **=118**	***n*** **=28**
Very Comfortable	4 (3)	2 (7)
Somewhat Comfortable	22 (19)	8 (29)
Neither Comfortable nor uncomfortable	22 (19)	4 (14)
Somewhat uncomfortable	32 (27)	1 (4)
Very uncomfortable	38 (32)	13 (46)

**Table 3:** IM resident and GIM faculty report on past clinical use of POCUS

	*n* (%)	
	Residents (n=104)	Faculty (n=19)
Central Line Insertion	79 (76)	8 (42)
Assessment of Ascites and Paracenteis	70 (67)	13 (68)
Assessment of Pleural Effusions and Thoracentesis	61 (59)	12 (63)
Echocardiography: Valvular Disease or Ejection Fraction	31 (30)	1 (5)
Detection of Pericardial Fluid	30 (29)	4 (21)
Arterial Line Insertion	28 (27)	1 (5)
Volume Assessment with IVC Measurement	14 (13)	1 (5)
Knee Arthrocentesis	1 (1)	0 (0)

Other Reported Clinical Uses of POCUS: Focused Abdominal Sonography for Trauma Rule out Pneumothorax Marking a Site for Lumbar Puncture Detection of Proximal Deep Vein Thrombosis		

**Table 4:** IM resident (*n*=116) and GIM faculty (*n*=29) opinion on most valuable POCUS uses for IM

	*n* (%)	
	Residents	Faculty
Central Line Insertion	107 (92)	25 (86)
Assessment of Pleural Effusions and Landmarking for Thoracentesis	103 (89)	28 (97)
Assessment of Ascites and Landmarking for Paracenteis	99 (85)	28 (97)
Detection of Pericardial Fluid	86 (74)	18 (62)
Echocardiography: Valvular Disease or Ejection Fraction	65 (56)	2 (7)
Volume Assessment with IVC Measurement	59 (51)	5 (17)
Arterial Line Insertion	35 (30)	2 (7)
Knee Arthrocentesis	32 (28)	8 (28)
Diagnosis of Abdominal Aortic Aneurysm	32 (28)	4 (14)

**Table 5:** Opinion on most effective educational course model for ultrasound training

	*n* (%)	
	Residents (*n*=115)	Faculty (*n*=28)
Combined Didactic and Practical	101 (86)	25 (89)
Self-teaching and Supervised Tutorials	12 (10)	1 (4)
On-line Teaching Modules	2 (2)	0 (0)
Other: “Practical Teaching” “Online Modules and Practical Teaching” “Procedural Rotation Mixed with POCUS Teaching from a Radiologist”	2 (2)	2 (7)

**Table 6:** Reported clinical applications of POCUS by national IM program leaders (*n* = 9)

	*n* (%)
POCUS guided vascular access	9 (100)
POCUS guided thoracentesis	6 (67)
POCUS guided paracentesis	5 (56)
Abdominal assessment (i.e. ascites)	3 (33)
Pulmonary assessment (i.e. pleural effusions)	2 (22)
Cardiac assessment (i.e. left ventricle function)	1 (11)
Integument assessment (i.e. abscess)	1 (11)
POCUS guided arthrocentesis	1 (11)
POCUS guided lumbar puncture	0 (0)
Vascular assessment (i.e. deep vein thrombosis)	0 (0)

**Table 7:** Perceived barriers by GIM program/division directors and core IM program directors to the introduction of POCUS in IM curricula (*n*= 15)

	*n* (%)
Lack of faculty trained in POCUS	13 (87)
Lack of access to a POCUS machine	7 (47)
Financial reasons	6 (40)
Formal radiology studies readily accessible	5 (33)
Opposition from other ultrasound trained physicians (radiologists/cardiologists)	4 (27)
Lack of interest/support from the department	4 (27)
Concerns that POCUS requires a long period of training	3 (20)
Other: “Curriculum Overload”	1 (7)

## Appendix A Local Survey, Resident Cohort

Question 1: Age

□ 19–24
□ 25–29
□ 30–35
□ 35–39
□ >40

Question 2: Gender

□ Male
□ Female

Question 3: Level of Training

□ ^*^PGY-1
□ PGY-2
□ PGY-3
□ PGY-4

^*^PGY – Post graduate year of residency

Question 4: Have you used ultrasound for diagnostic or therapeutic purposes previously?

□ More than 10 independent ultrasounds without help or guidance
□ Witnessed multiple ultrasounds, more than 5 independent ultrasounds without help or guidance
□ Witnessed ultrasounds performed and have brief exposure with expert assistance
□ Witnessed ultrasounds, but never personally performed one
□ Never witnessed or used an ultrasound machine

Question 5: Have you used ultrasound on a General Medicine rotation previously?

□ Yes, please specify __________
□ No

Question 6: Have you used ultrasound previously on a subspecialty rotation?

□ Yes, please specify __________
□ No

Question 7: What have you used ultrasound for? (Choose all that apply)

□ Central line insertion
□ Arterial line insertion
□ Assessment of pleural effusion and thoracentesis
□ Assessment of ascites and paracentesis
□ Echocardiography: valvular disease or ejection fraction
□ Detection of pericardial fluid
□ Knee arthrocentesis
□ Volume assessment with IVC measurements
□ Other, please specify: __________

Question 8: Did you receive any previous training in sonography?

□ Yes, I have received formal training in sonography in general
□ Yes, I have received formal training in performing sonography on a specific procedure
□ Yes, I have received informal training on performing specific ultrasound-guided procedure
□ No, I have not had any training in sonography

Question 9: Do you feel comfortable now with the use of ultrasound for procedures?

□ Very comfortable
□ Somewhat comfortable
□ Neither comfortable nor uncomfortable
□ Somewhat uncomfortable
□ Very uncomfortable

Question 10: Do you think having ultrasound skills is relevant for Internal Medicine residents?

□ Very relevant
□ Somewhat relevant
□ Neither relevant or irrelevant
□ Somewhat irrelevant
□ Very irrelevant

Question 11: What do you think will be the most valuable application of ultrasound for Internal Medicine residents? (choose all that apply)

□ Central line insertion
□ Arterial line insertion
□ Assessment of pleural effusion and thoracentesis
□ Assessment of ascites and paracentesis
□ Echocardiography: valvular disease or ejection fraction
□ Detection of pericardial fluid
□ Knee arthrocentesis
□ Volume assessment with IVC measurements
□ Diagnosis of abdominal aortic aneurysm
□ Other, please specify: __________

Question 12: Are you interested in receiving training in sonography as part of the Internal Medicine residency program?

□ Very interested
□ Somewhat interested
□ Equivocal
□ Not interested

Question 13: What do you think is the most effective educational course model for ultrasound training?

□ Combined didactic and practical
□ Self-teaching and supervised tutorials
□ On-line teaching module
□ Other, please specify: __________

Question 14: At what level of training should ultrasound be introduced?

□ Medical School
□ PGY1
□ PGY2
□ PGY3
□ Not at all

Question 15: Where do you think is the best venue for teaching ultrasound to Toronto’s Internal Medicine residents?

□ PGME (Post Graduate Medical Education) core session
□ A CRISP (Core Resident Integrated Scholarly Program) session
□ “Procedure day” on ultrasound training
□ A weekend training session on ultrasound
□ One week of ultrasound training in the first year of IM training
□ Other, please specify: __________

Question 16: Do you give consent for the information in this survey to be used for research purposes?

□ Yes
□ No

## Appendix B Local Survey, Staff Physician Cohort

Question 1: Time since FRCP certification

□ 1–5 years
□ 5–10 years
□ 10–20 years
□ >20 years

Question 2: Gender

□ Male
□ Female

Question 3: Have you used ultrasound for diagnostic or therapeutic purposes previously?

□ More than 10 independent ultrasounds without help or guidance
□ Witnessed multiple ultrasounds, more than 5 independent ultrasounds without help or guidance
□ Witnessed ultrasounds performed and have brief exposure with expert assistance
□ Witnessed ultrasounds, but never personally performed one
□ Never witnessed or used an ultrasound machine

Question 4: Have you used ultrasound on General Medicine service previously?

□ Yes
□ No

Question 5: What have you used ultrasound for? (choose all that apply)

□ Central line insertion
□ Arterial line insertion
□ Assessment of pleural effusion and thoracentesis
□ Assessment of ascites and paracentesis
□ Echocardiography: valvular disease or ejection fraction
□ Detection of pericardial fluid
□ Knee arthrocentesis
□ Volume assessment with IVC measurements
□ Other, please specify: __________

Question 6: Did you receive any previous training in sonography?

□ Yes, I have received formal training in sonography in general
□ Yes, I have received formal training in performing sonography on a specific procedure
□ Yes, I have received informal training on performing specific ultrasound-guided procedure
□ No, I have not had any training in sonography

Question 7: Do you feel comfortable now with the use of ultrasound for procedures?

□ Very comfortable
□ Somewhat comfortable
□ Neither comfortable nor uncomfortable
□ Somewhat uncomfortable
□ Very uncomfortable

Question 8: Do you think having ultrasound skills is relevant for Internal Medicine residents?

□ Very relevant
□ Somewhat relevant
□ Neither relevant or irrelevant
□ Somewhat irrelevant
□ Very irrelevant

Question 9: What do you think will be the most valuable application of ultrasound for Internal Medicine residents? (choose all that apply)

□ Central line insertion
□ Arterial line insertion
□ Assessment of pleural effusion and thoracentesis
□ Assessment of ascites and paracentesis
□ Echocardiography: valvular disease or ejection fraction
□ Detection of pericardial fluid
□ Knee arthrocentesis
□ Volume assessment with IVC measurements
□ Diagnosis of abdominal aortic aneurysm
□ Other, please specify: __________

Question 10: Are you interested in receiving training in sonography?

□ Very interested
□ Somewhat interested
□ Equivocal
□ Not interested

Question 11: How would you like to receive sonography training?

□ Self-directed online module
□ A local weekend of didactic and practical training sessions
□ A weekend course at a centre of excellence
□ Other, please specify: __________

Question 12: Are you interested in including training in sonography as part of the Internal Medicine residency program?

□ Very interested
□ Somewhat interested
□ Equivocal
□ Not interested

Question 13: What do you think is the most effective educational course model for ultrasound training?

□ Combined didactic and practical
□ Self-teaching and supervised tutorials
□ On-line teaching module
□ Other, please specify: __________

Question 14: At what level of training should ultrasound be introduced?

□ Medical School
□ PGY1
□ PGY2
□ PGY3
□ Not at all

Question 15: Where do you think is the best venue for teaching ultrasound to Toronto’s Internal Medicine residents?

□ PGME (Post Graduate Medical Education) core session
□ A CRISP (Core Resident Integrated Scholarly Program) session
□ “Procedure day” on ultrasound training
□ A weekend training session on ultrasound
□ One week of ultrasound training in the first year of IM training
□ Other, please specify: __________

Question 16: Do you give consent for the information in this survey to be used for research purposes?

□ Yes
□ No

## Appendix 3 National Survey

Question 1: Do you give consent for the information in this survey to be used for research purposes?

□ Core Internal Medicine Program Director
□ General Internal Medicine Division Head
□ General Internal Medicine R4 Program Director
□ Other, please specify: __________

Question 2: Is point-of-care ultrasound (POCUS) incorporated into your GIM Training Program or GIM fellowship (excluding non-GIM fellowship programs such as cardiology)?

□ Yes
□ No

Question 3: If yes, please specify the usage of POCUS at your site (choose all that apply):

□ Cardiac (e.g. for the assessment of LV function)
□ Pulmonary (e.g. for the assessment of Pleural effusions)
□ Abdominal (e.g. for the assessment of Ascites)
□ Vascular (e.g. for the assessment of DVT assessment)
□ Integument (e.g. for the assessment of abscesses)
□ POCUS guided vascular access
□ POCUS guided thoracentesis
□ POCUS guided paracentesis
□ POCUS guided arthrocentesis
□ POCUS guided lumbar punctures
□ Other, please specify: __________

Question 4: As part of your Internal Medicine program or GIM fellowship, does your curriculum include formal training on POCUS?:

□ Yes
□ No

Question 5: If yes, please elaborate on the type and amount of training provided (if none, please choose “0 hours”:

**Table:**

	0 hours	< 1 hour	1–10 hours	> 10 hours
Didactic teaching	□	□	□	□
Ultrasound Image or Video training	□	□	□	□
Hands on training with simulators	□	□	□	□
Hands on training with patients	□	□	□	□
Case logs	□	□	□	□
Other, please specify: __________				

Question 6: In your opinion, should POCUS be used by residents and staff physicians on the General Internal Medicine service?

□ Strongly Agree
□ Agree
□ Neutral
□ Disagree
□ Strongly Disagree

Question 7: Regarding Access to POCUS, does your department:

□ Own a dedicated machine for GIM/CIM?
□ Have access to another machine?
□ Neither own nor have access to a machine?

Question 8: At your centre, what are the obstacles to the introduction of POCUS?

□ Lack of access to a POCUS machine
□ Lack of Faculty trained in POCUS
□ Lack of interest/support from the department
□ Formal radiology studies readily accessible
□ Financial reasons
□ Opposition from other US-trained physicians (radiologist/cardiologists)
□ Concerns that POCUS requires a long period of training
□ Other, please specify: __________

Question 9: Please add any additional comments:

□ __________

## References

[1] Moore CL, Copel JA (2011). Point-of-care ultrasonography. New England J Med.

[2] Duvall WL, Croft LB, Goldman ME (2003). Can hand-carried ultrasound devices be extended for use by the noncardiology medical community. Echocardiography.

[3] Decara JM, Lang RM, Spencer KT (2003). The hand-carried echocardiographic device as an aid to the physical examination. Echocardiography.

[4] Osranek M, Bursi F, O’Leary PW (2003). Hand-carried ultrasound-guided pericardiocentesis and thoracentesis. J Am Soc Echocardiog.

[5] Vignon P, Dugard A, Abraham J (2007). Focused training for goal-oriented hand-held echocardiography performed by noncardiologist residents in the intensive care unit. Intens Care Med.

[6] Dodge KL, Lynch CA, Moore CL (2012). Use of ultrasound guidance improves central venous catheter insertion success rates among junior residents. J Ultras Med.

[7] Kane D, Grassi W, Sturrock R, Balint PV (2004). Musculoskeletal ultrasound-a state of the art review in rheumatology. Part 2: Clinical indications for musculoskeletal ultrasound in rheumatology. Rheumatology.

[8] Patil P, Dasgupta B (2012). Role of diagnostic ultrasound in the assessment of musculoskeletal diseases. Ther Adv Musculoskelet Dis.

[9] Noble VE, Lamhaut L, Capp R (2009). Evaluation of a thoracic ultrasound training module for the detection of pneumothorax and pulmonary edema by prehospital physician care providers. BMC Med Educ.

[10] Nass K, O’Neill WC (1999). Bedside renal biopsy: ultrasound guidance by the nephrologist. Am J Kidney Dis.

[11] Brennan JM, Ronan A, Goonewardena S (2006). Handcarried ultrasound measurement of the inferior vena cava for assessment of intravascular volume status in the outpatient hemodialysis clinic. Clin J Am Soc Nephro.

[12] (2008). Emergency ultrasound guidelines.

[13] Socransky S (2006). Emergency department targeted ultrasound: 2006 update. Can J Emerg Med.

[14] Shackford SR, Rogers FB, Osler TM (1999). Focused abdominal sonogram for trauma: the learning curve of nonradiologist clinicians in detecting hemoperitoneum. Trauma.

[15] Nguyen VTQ, Ho JE, Ho CY, Givertz MM, Stevenson LW (2008). Handheld echocardiography offers rapid assessment of clinical volume status. Am Heart J.

[16] Brennan JM, Blair JE, Goonewardena S (2007). Comparison by medicine residents of physical examination versus hand-carried ultrasound for estimation of right atrial pressure. Am J Cardiol.

[17] Royse CF, Seah JL, Donelan L, Royse AG (2006). Point of care ultrasound for basic haemodynamic assessment: novice compared with an expert operator. Anaesthesia.

[18] Croft LB, Duvall WL, Goldman ME (2006). A pilot study of the clinical impact of hand-carried cardiac ultrasound in the medical clinic. Echocardiography.

[19] Alexander JH, Peterson ED, Chen AY, Harding TM, Adams DB, Kisslo JA (2004). Feasibility of point-of-care echocardiography by internal medicine house staff. Am Heart J.

[20] Blaivas M (2005). Emergency diagnostic paracentesis to determine intraperitoneal fluid identity discovered on bedside ultrasound of unstable patients. J Emerg Med.

[21] Mynarek G, Brabrand K, Jakobsen JA, Kolbenstvedt A (2004). Complications following ultrasound-guided thoracocentesis. Acta Radiologica.

[22] Pihlajamaa K, Bode MK, Puumalainen T, Lehtimäki A, Marjelund S, Tikkakoski T (2004). Pneumothorax and the value of chest radiography after ultrasound-guided thoracocentesis. Acta Radiologica.

[23] Josephson T, Nordenskjold CA, Larsson J, Rosenberg LU, Kaijser M (2009). Amount drained at ultrasound-guided thoracentesis and risk of pneumothorax. Acta Radiologica.

[24] Patel PA, Ernst FR, Gunnarsson CL (2012). Evaluation of hospital complications and costs associated with using ultrasound guidance during abdominal paracentesis procedures. J Med Econ.

[25] Mehta N, Valesky WW, Guy A, Sinert R (2013). Systematic review: is real-time ultrasonic-guided central line placement by ED physicians more successful than the traditional landmark approach?. Emerg Med J.

[26] Wiler JL, Costantino TG, Filippone L, Satz W (2010). Comparison of ultrasound-guided and standard landmark techniques for knee arthrocentesis. The J Emerg Med.

[27] Wong BM, Ackroyd-Stolarz S, Bukowskyj M (2014). The CanMEDS 2015 Patient Safety and Quality Improvement Expert Working Group Report.

[28] Keddis M, Cullen M, Reed D (2011). Effectiveness of an ultrasound training module for internal medicine residents. BMC Med Educ.

[29] Riegert-Johnson D, Bruce C, Montori V (2005). Residents can be trained to detect abdominal aortic aneurysms using personal ultrasound imagers: a pilot study. J Am Soc Echocardiog.

[30] (2013). Position Statement on the Use of Point of Care Ultrasound.

[31] Arishenkoff S, Blouw M, Card S (2014). Expert Consensus on a Canadian Internal Medicine Ultrasound Curriculum. Can J Gen Int Med.

[32] Johnson LC, Beaton R, Murphy S, Pike K (2000). Sampling bias and other methodological threats to the validity of health survey research. Int J Stress Manage.

